# A preliminary study for the clinical effect of one combinational physiotherapy and its potential influence on gut microbial composition in children with Tourette syndrome

**DOI:** 10.3389/fnut.2023.1184311

**Published:** 2023-09-15

**Authors:** Chun Bao, Meng Wei, Hongguo Pan, Ming Wen, Ziming Liu, Yue Xu, Huihui Jiang

**Affiliations:** ^1^Department of Child Healthcare, Xiangyang No. 1 People’s Hospital, Hubei University of Medicine, Xiangyang, China; ^2^Zhangjiang Center for Translational Medicine, Shanghai Biotecan Pharmaceuticals Co., Ltd., Shanghai, China

**Keywords:** Tourette syndrome, gut microbiota, cranial electrotherapy stimulation, electrodermal biofeedback training, combinational physiotherapy

## Abstract

**Introduction:**

Tourette syndrome (TS) is a chronic neuropsychiatric disorder with unknown causes and inadequate therapies. Inspired by the important roles of gut microbiota in some mental illnesses, the interactions between gut microbiota and TS via the gut-brain axis have gained more and more attention. This study aimed to characterize the gut microbial profiles in children with TS, and explore the clinical effects of one combinational physiotherapy and its potential influence on gut microbial composition.

**Methods:**

The gut microbial profiles were depicted based on the sequence data of 32 patients and 29 matched health children by 16S rDNA amplicon pyrosequencing. Thirty of thirty-two patients underwent uninterrupted two 10-day courses of combinational physiotherapy, which included a 60-minute cranial electrotherapy stimulation (CES) training followed by a 30-minute biofeedback training per session, 2 sessions a day.

**Results:**

Our results indicated that the gut microbial composition in children with TS was different from that in healthy controls. Multiple GBM neurotransmitter modules obtained through Picrust2 functional predictive analysis were significantly increased in patients, including Histamine degradation, Dopamine degradation, and DOPAC synthesis. Moreover, this combinational physiotherapy could significantly diminish tic activity, whose positive effects were first reported in children with TS. Lastly, different gut microbial compositions and predictive metabolic pathways were also observed between patients before and after this treatment, with lower abundances of the genera (e.g., *Dorea*) and significant decreases of GBM neurotransmitter modules (e.g. dopamine degradation) in patients after this treatment, indicating that improved clinical symptoms might be accompanied by an improvement of intestinal microenvironment.

**Discussion:**

Children with TS showed a cognizable gut microbial profile, and certain enriched bacteria with pro-inflammatory potential might induce neuroinflammatory responses. This combinational physiotherapy could significantly diminish tic activity, and the gut microbial compositions in patients after this treatment were different from those without any treatment, indicating the existence of bidirectional communication of the gut-brain axis in TS. But studies on the gut microbial characteristics in TS patients, the influences of gut microbiota on tic severity, the efficacy and safety of this treatment, and the bidirectional regulatory mechanism between brain signals and gut microbiota in TS still need to be explored.

## Introduction

Tourette syndrome (TS) is a chronic neurological disease, whose symptoms include persisting multiple motor tics and alternating vocal tics (1). Population studies indicated that the prevalence of TS in children is about 1.7% in China and 0.8% worldwide (2, 3). Despite most patients can recover by themselves before the age of 18, approximately 33% of patients will continue their symptoms to adulthood factually (4).

Currently, although the cause of TS is unknown, genetic and environmental factors are regarded as substantial factors associated with its intrinsic etiologies. Many studies indicated that the development and regulation of the brain are affected by the gut microbiota through the microbiota-gut-brain axis (5, 6). In patients with tic disorders (TDs), the abundances of *Bacteroides plebeius* and *Ruminococcus lactaris* were increased compared to healthy children, while *Prevotella stercorea* and *Streptococcus lutetiensis* were decreased (7). Among these patients, a significant enrichment of *Ruminococcus lactaris* was identified in patients with TS than that in patients with other TDs (7). What’s more, *Klebsiella pneumonia*, as a GABA-degrading bacterium, was positively associated with the symptomatic deterioration in TDs (8). Meanwhile, *Eubacterium* spp., *Bifidobacterium* spp., and *Akkermansia muciniphila* were related to the production of GABA, which showed a negative correlation with the Yale Global Tic Severity Scale (YGTSS) score (8, 9). However, some inconsistencies also existed. For example, as GABA-producing bacteria, *Bacteroides thetaiotaomicron* and *Bacteroides eggerthii* displayed a positive correlation with the YGTSS score (7). Thus, what’s kind of abnormal alterations of gut microbiota in TS and whether these abnormal alterations were the key pathogenic factors still need to be explored.

Treatments of TS mainly include drug treatment and psychological treatment. However, both therapies could not completely relieve the clinical symptom and had their limitations, such as side effects from medication and a long treatment period for psychological treatment (10, 11). In addition, although some noninvasive techniques (e.g., transcranial magnetic stimulation or transcranial direct current stimulation) presented positive results for TS, their wide clinical applications were limited due to the high price (12, 13).

As a noninvasive transcutaneous therapeutic device, cranial electrotherapy stimulation (CES) adopted pulsed, alternating microcurrent (<1,000 μA) to the brain depending on its electrodes placed on the earlobes, mastoid processes, zygomatic arches, or the maxillo-occipital junction. CES has been approved by Food and Drug Administration (FDA) to treat patients with depression, anxiety, insomnia or pain, and it is relatively inexpensive and convenient compared with other noninvasive stimulation means (14–16). Although several studies have reported the efficacy and safety of CES in children with TS (17–19), its effectiveness using alone was less and slower in comparison with aripiprazole for children and adolescents with TS (20). Meanwhile, as a noninvasive psychophysiological intervention, biofeedback can regulate patients’ physiological responses by allowing patients to perceive visual and auditory signals. The tic frequency in patients with TS was significantly decreased during relaxation biofeedback compared to arousal biofeedback, and it was positively correlated with sympathetic arousal during the sessions of arousal biofeedback (21). However, another study found that maintaining longstanding relaxation biofeedback was difficult because of the concomitant occurrence of tics, even though the tic frequency was reduced during 5 min of relaxation biofeedback (22).

Until now, the brain and the gut communicate with each other via various routes, such as the immune system, tryptophan metabolism, vagus nerve and enteric nervous system (6, 23). Many earlier studies regarding gut-brain communication focused on digestive function and satiety (24–26), whereas recently growing works have concentrated on higher-order cognitive and psychological effects of this bidirectional communication (27–29). As a chronic neurological disease, certain gut microbial alterations have been observed in TS. However, little information about the gut microbial composition after the clinical improvement in TS patients was reported. Zhao et al. have reported that tic symptoms ameliorated notably after 8 weeks of fecal microbiota transplantation (FMT) for patients with TS, and the gut microbial composition was significantly altered, especially with the restoration of *Bacteroides coprocola* (30, 31). In the TS mice model, the symptoms were dramatically improved after receiving feces from healthy mice for 3 weeks, and the abundances of *Turicibacteraceae* and *Ruminococcaceae* were significantly increased in their feces (32). In addition, the treatment of acupuncture and massage was effective for children with TS, whose gut microbial characteristics after the treatment was close to those in healthy children (33). Noteworthy, in addition to diet and drugs, other undiscovered mechanisms also might have potential influences on the distribution of gut microbiota. On the other hand, the occurrence of gut microbial alterations is reasonable when the clinical symptoms associated with emotion ameliorated dramatically, partly due to the bidirectional communication between the gut and the brain.

The aims of this study were as follows: (1) to characterize the gut microbial distribution in children with TS; (2) to explore the clinical effects of one combinational physiotherapy on children with TS; (3) to explore the potential impact of the combinational physiotherapy on gut microbial composition.

## Materials and methods

### Ethical approval

This study was approved by the Ethics Committee of the Xiangyang No. 1 People’s Hospital, Hubei University of Medicine (Xiangyang, China). The corresponding Institutional Review Board (IRB) number was No.2020KYLL. All written informed consents were obtained from all subjects’ guardians.

### Subjects

Thirty-two children with TS who visited the Department of Child Healthcare, Xiangyang No. 1 People’s Hospital, Hubei University of Medicine from June 2021 to July 2022 were recruited for this study. The patients were aged 2.92–13 years, with a median age with IQR being 7.00 (5.27–9.75) years (Table 1). Twenty-six of thirty-two children were male and six were female. The inclusion criteria were as follows: (1) diagnosed as TS by a comprehensive evaluation according to the Expert Consensus on the Diagnosis and Treatment of Tic Disorders in Children (2017 Practical Edition) and the 5th edition of the Diagnostic and Statistical Manual of Mental Disorders (DSM-5); (2) diagnosed for the first time not receiving any treatments. The exclusion criteria consisted of the following: (1) the presence of intellectual disability, autism, tristimania, manic-depressive psychosis, epilepsy, Parkinson’s disease, schizophrenia, chorea, athetosis, and other nervous system diseases; (2) a history of hepatolenticular degeneration and other extra vertebral diseases; (3) a history of obesity, gastrointestinal disease, gastrointestinal injury, head injury, and other physical illnesses; (4) other diseases which were unsuitable for this study evaluated by our investigators; (5) the experience of glucocorticoids, immune-suppressants, antihistamines, and other drugs for neurological diseases within the last year; (6) the experience of antibiotics, probiotics, or prebiotics within 3 months before sampling. The diagnosis was made by three independent neurological clinicians. Twenty-nine children were recruited as healthy controls during the same period, including 24 males and 5 females. All healthy controls underwent health checkups in our hospital to exclude any physical illnesses, mental diseases, or the above-mentioned experiences. The healthy children were aged 2.75–14 years, with a median age with IQR being 6.42 (4.75–8.59) years (Table 1). In addition, all recruited subjects’ mothers did not take probiotics or prebiotics during pregnancy.

**Table 1:** Demographic and clinical characteristics of children with and without Tourette syndrome (TS).

Clinical characteristics	No. of patients	Groups		*p* value
		Healthy controls	TS	
Total Sample	61	29	32	
Gender				
Male	50	24	26	0.878
Female	11	5	6	
Premature birth				
Yes	4	1	3	0.350
No	57	28	29	
Natural labour				
Yes	19	10	9	0.592
No	42	19	23	
Breast feeding				
Yes	48	23	25	0.910
No	13	6	7	
Gestational stress				
Yes	7	4	3	0.589
No	54	25	29	
The history gestational infections				
Yes	0	0	0	1.000
No	61	29	32	
Taking antiemetic drugs during pregnancy				
Yes	5	5	0	0.014^*^
No	56	24	32	
Smoking history during pregnancy				
Yes	0	0	0	1.000
No	61	29	32	
Drinking history during pregnancy				
Yes	0	0	0	1.000
No	61	29	32	
First baby for mother				
Yes	45	23	22	0.349
No	16	6	10	
Singleton pregnancy				
Yes	57	26	31	0.255
No	4	3	1	
The history of TS				
Yes	3	0	3	0.096
No	57	28	29	
Unknown	1	1	0	
Age/year (median with IQR)	7.00 (5.00–9.00)	6.42 (4.75–8.59)	7.00 (5.27–9.75)	0.495
Height/m (median with IQR)	1.28 (1.18–1.40)	1.23 (1.10–1.40)	1.30 (1.20–1.40)	0.365
Weight/kg (median with IQR)	27.00 (20.50–32.00)	25.00 (19.50–32.00)	27.00 (21.25–33.50)	0.448
BMI/(kg/m^2^) (median with IQR)	16.12 (14.73–17.78)	16.23 (14.72–18.64)	15.99 (14.74–17.51)	0.471

^*^p < 0.05.

### Collection of clinical data

Information about the children’s general condition and pregnancy-related condition was collected, including the date of TS onset, first symptoms, current symptoms, symptom frequency, daily activities, learning and social situations, feeding option, gestational stress, gestational infectious history, and others. The Yale Global Tic Severity Scale (YGTSS) is a clinician-rated evaluation of patients’ tic severity over the past 7–10 days. Briefly, a clinician directly interviews each child and their guardian to produce the Total Motor and Phonic Tic score [range 0–50, including the separate Total Motor Tic score (range 0–25) and the separate Total Phonic Tic score (range 0–25)] and the separate tic-related impairment score (range 0–50). Specifically, 46 tic disorder symptoms are involved in the YGTSS, including 12 simple motor tics (e.g., eye blinking), 19 complex motor tics (e.g., facial expressions), seven simple vocal tics (e.g., coughing), and eight complex vocal tics (e.g., words), with unmentioned symptoms labeled as “other” symptoms in patients’ medical records. The motor and phonic domains are evaluated separately on a 0–5 scale across 5 dimensions (number, frequency, intensity, complexity, and interference), but the same anchor point descriptions are adopted to guide their scoring. For categorizing the severity degree of TS, the scores of 0–25, 26–50, and 51 or above are suitable for the determinations of mold, moderate, and severe stages, respectively.

### Combinational physiotherapy intervention

CES Therapy---As our selected CES device, the fifth generation alpha-stim stress control system was purchased from Electromedical Products International (Mineral Wells, Tex), and its 510(K) Number was K903014 approved by FDA. This alpha-stim stress control system could generate bipolar, asymmetric rectangular waves with a frequency of 0.5 Hz and a current intensity ranging from 0 μA to 500 μA. Before treatment, two ear clip electrodes were placed on the child’s right and left earlobes. The current intensity was adjusted until the child felt a mild tingling sensation and/or dizziness, and then the selected current intensity was reduced to slightly below the threshold of sensation. For one patient, the established current intensity will be consistently used throughout the whole course of the twenty-day treatment.

Biofeedback training---The multichannel biofeedback apparatus SPIRIT-2/4/8 was provided by the manufacturer, SPIRIT (Chengdu, China), and its Registration Certificate Number was Sichuan machine registration 20,182,260,085. The procedure of biofeedback training followed the product specification. Five dry nickel-plated electrodes (about 33 × 46 mm^2^ area per nickel-plated electrode) were placed on the child’s tic sites. The sampling rate was 256–1,024 times per second. Biofeedback was adopted as a form of computer-generated animation and displayed on the computer screen. Every child lay on a comfortable chair in front of the computer monitor to receive treatment. When the child felt relaxed and his/her skin conductance was lower than the set threshold, the videos were continuously playing; if the patients’ skin conductance increased and exceeded the set threshold, the videos would get smaller until to disappear. Thus, our biofeedback therapy “rewarded” children for controlling or even reducing their electrodermal sympathetic activity by displaying more animations.

During the uninterrupted two 10-day course of treatment, the children attended a 60-min CES training followed by a 30-min biofeedback training per session, 2 sessions a day, a total of 40 sessions. Notably, the interval between two sessions per day was 4 h. During each session, a nurse instructed the children to participate in the process, and adjust the animation by encouraging them to relax both mentally and physically. Children could not fall asleep during treatment.

The therapeutic effect was evaluated at the end of each course for every patient. The Y-GTSS was used to evaluate patients’ tic severity by their attending physicians, and the side effects were also recorded in patients’ electronic medical records.

### Collection of fecal specimens

All recruited patients were encouraged to collect their feces within 2 days before (TS-pre) and after (TS-post) the combinational physiotherapy. Fecal specimens (about soybean grain size, two pieces per time) from every child were collected by themselves or their guardians at home or hospital within 3 min after defecating. After sampling, the fecal samplers (Biotecan, Shanghai, China) were sealed, labeled, and transferred (<18°C) to Biotecan Laboratories within 2 days and stored at −80°C.

### 16S rRNA gene sequencing and data processing

A total of 78 fresh feces samples (HC vs. TS-pre vs. TS-post, 29 vs. 32 vs. 17) were collected in fecal samplers and stored at −80°C until being used to perform high-throughput sequencing. The QIAamp PowerFecal Pro DNA Kit (QIAGEN, Germany) was adopted to extract bacterial genomic DNA, which was amplified by Phusion High-Fidelity PCR Master Mix (New England Biolabs, Massachusetts State, United States) targeting the V3V4 region of 16S rRNA genes (Forward primer: 341F 5′-CCTACGGGNGGCWGCAG-3′, Reverse primer: 805R 5′-GACTACHVGGGTATCTAATCC-3′). The PCR products were purified by the TransStart® FastPfu DNA Polymerase kit (TransGen, Beijing, China). The DNA quantification of the purified product was finished by the Qubit dsDNA HS Assay Kit (Thermo Fisher Scientific, Massachusetts State, United States). A library Quant Kit Illumina GA revised primer-SYBR Fast Universal (KAPA Biosystems, Massachusetts, United States) was adopted to perform library quantification, and then a Novaseq6000 500 cycle (Illumina, California, United States) was used to perform pair-end 2 × 250 bp sequencing.

To cope with these sequencing data, the Quantitative Insights Into Microbial Ecology 2 (QIIME 2, v2017.6.0) pipeline and previous criteria were adopted (34, 35). Vsearch V2.4.4 was used to assemble the paired-end reads (36). 16S rRNA gene sequences were assigned to operational taxonomic units (OTUs) according to a similarity cutoff value of 97%, which was against the Greengenes database by Vsearch V2.4.4. Notable, OTUs with <0.001% entire sequences were dumped. The final OTU table was averaged, rounded, and rarefied, whose generation was based on averaging 100 evenly resampled OTU subsets under 90% of the minimum sequencing depth. Abundance curves were arranged by OTU level. The sequencing depth was evaluated and determined via rarefaction analysis.

### Bioinformatics and statistical analyses

The chi-square test was adopted to analyze the statistical differences in categorical variables between healthy controls and patients with TS in SPSS 23.0 (IBM, Chicago, IL, United States). Continuous variables were presented as median with interquartile range (IQR) and compared by Mann–Whitney ***U***-test between groups in GraphPad Prism version 7.0 (GraphPad, San Diego, CA, United States). Y-GTSS scores between patients before and after the combinational physiotherapy were displayed as mean with SD and compared by Wilcoxon matched-pairs signed rank test in GraphPad Prism version 7.0. Venn diagram, heat-map analysis, and correlation analysis were performed by R software (v3.6.3). GraPhlAn^1^ was used to depict the phylogenetic tree.

Alpha diversity analysis, including the Chao1 index, Simpson index, and Shannon index, was constructed by QIIME 2, whose comparison adopted the Pairwise Wilcox test. Bray-Curtis distance metrics and visualized via principal coordinate analysis (PCoA) were used to carry out the beta diversity analysis. Gut microbial composition and structure were compared between groups using Permutational multivariate analysis of variance (PERMANOVA) via the Kruskal test in R software. The comparability between these two groups was assessed by a one-way analysis of similarities (Anosim) analysis. Linear discriminant analysis effect size (LEfSe) was used to identify abundant taxa with significant differences across groups based on the default parameters (logarithmic LDA score = 2) (37). Phylogenetic investigation of communities by reconstruction of unobserved states (PICRUSt, PICRUSt2 v2.3.0-b) was adopted to forecast gut microbial functions by annotating the gene catalog according to the Kyoto Encyclopedia of Genes and Genomes (KEGG) modules, carbohydrate-active enzymes (CAZY) database, MetaCyc database, GMM metabolic modularization, and GBM neurotransmitter modules enrichment analysis (38), whose statistical differences between groups were analyzed by Univar Test.

## Results

### Characteristics of subjects

TS-pre group consisted of 26 males and 6 females with a median age of 7.00 years (range: 2.92–13; interquartile range, IQR: 5.27–9.75) and a median BMI of 15.99 kg/m^2^ (range: 13.19–24.49; interquartile range, IQR: 14.74–17.51) (Table 1). According to clinical evaluation, all patients were diagnosed with mild severity with mean YGTSS scores of 34.97 (SD 5; range 28–45). Except for taking antiemetic drugs during pregnancy (*p* = 0.014), no demographic differences were observed between the TS-pre and HC groups in age, Height, Wight, BMI, gender, premature birth, natural labor, breastfeeding, gestational stress, gestational infections history, smoking history, drinking history, first baby for mother, singleton pregnancy, and the family history of TS (Table 1).

### Diversity and composition of gut microbiota in children with TS

**Figure 1:**
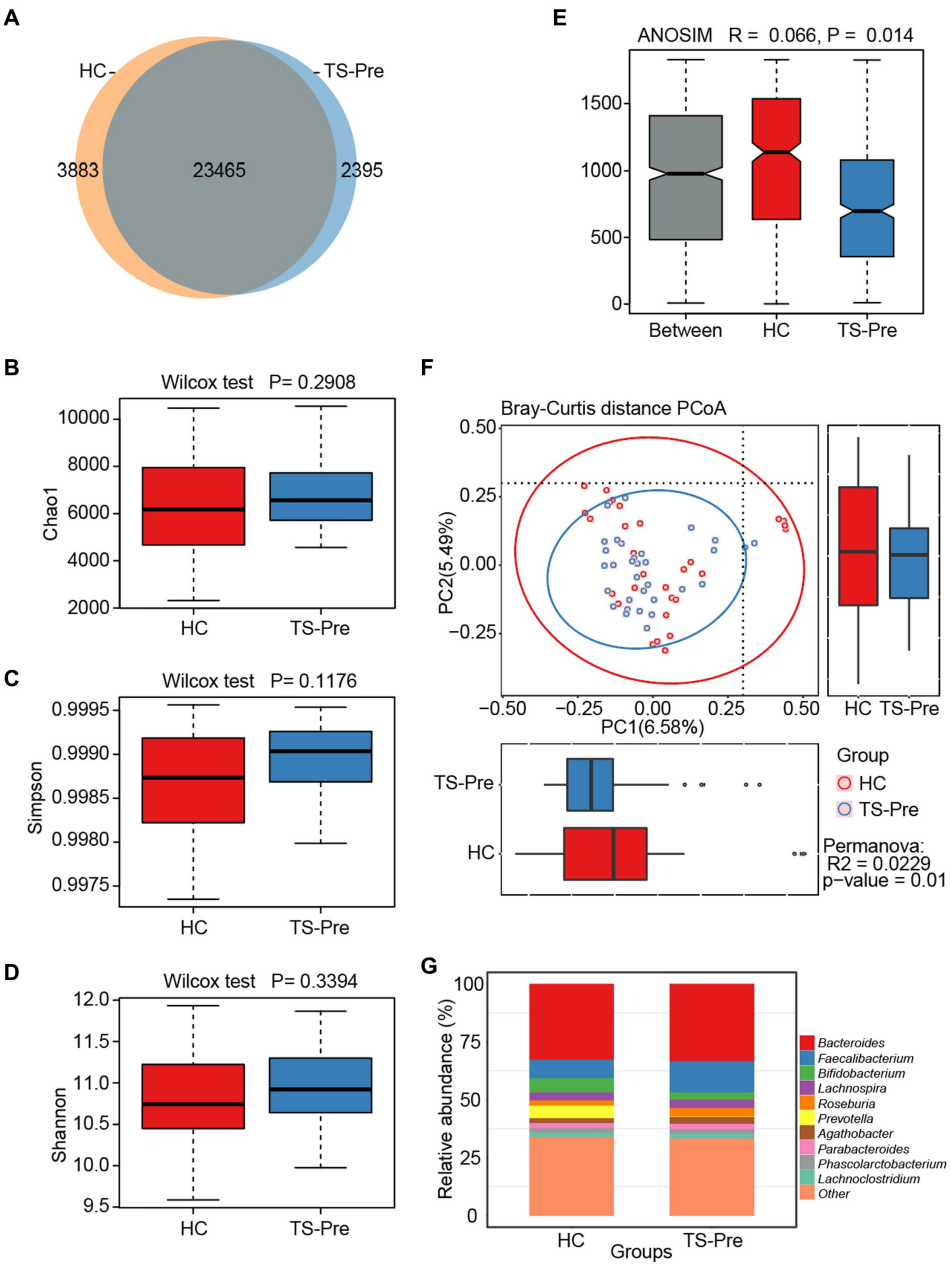
Diversity and composition of gut microbiota in healthy controls (HC, *n* = 29) and treatment-naive children with TS (TS-Pre, *n* = 32). **(A)** 23,465 OTUs were shared between groups. The HC group had the unique OTUs with 3,883, while the TS-Pre group had 2,395. Chao1 **(B)**, Simpson **(C)**, and Shannon **(D)** were used to analyze the alpha diversity between the HC group and the TS-Pre group. **(E)** Analysis of similarity (ANOSIM) presented significant differences between groups (*p* < 0.05). **(F)** The beta diversity between both groups was analyzed by principal coordinate analysis (PCoA) of the weighted UniFrac distance. **(G)** The relative abundance histograms of all genera in the HC and TS-Pre groups. The top 10 shared genera with high relative abundance were displayed by different colors and the remaining genera with less relative abundance were classified as other.

In the Venn diagram, the shared OTUs were 23,465 between the HC and TS-Pre groups, and the HC group had more unique OTUs with 3,883 than the TS-Pre group with 2,395 (Figure 1A). In α-diversity, no differences in gut microbial richness and evenness were identified between groups (Chao1 *p* = 0.2908, Simpson *p* = 0.1176, and Shannon *p* = 0.3394) (Figures 1B–D). In β-diversity, the difference between groups was more significant than within one group (*R* = 0.066, *p* < 0.05) (Figures 1E,F). Figure 1G and Supplementary Figure S1 displayed microbial composition at the genus level. *Bacteroides, Faecalibacterium, and Roseburia* were the major components in the TS-Pre group, whereas *Bacteroides, Faecalibacterium,* and *Bifidobacterium* in the HC group. *Faecalibacterium, Roseburia,* and *Agathobacter* were present in both groups, but were more predominant in the TS-Pre group. In contrast, although *Bifidobacterium* and *Prevotella* were also observed in these two groups, they were more prominent in the HC group.

### Marker genera in children with TS

**Figure 2:**
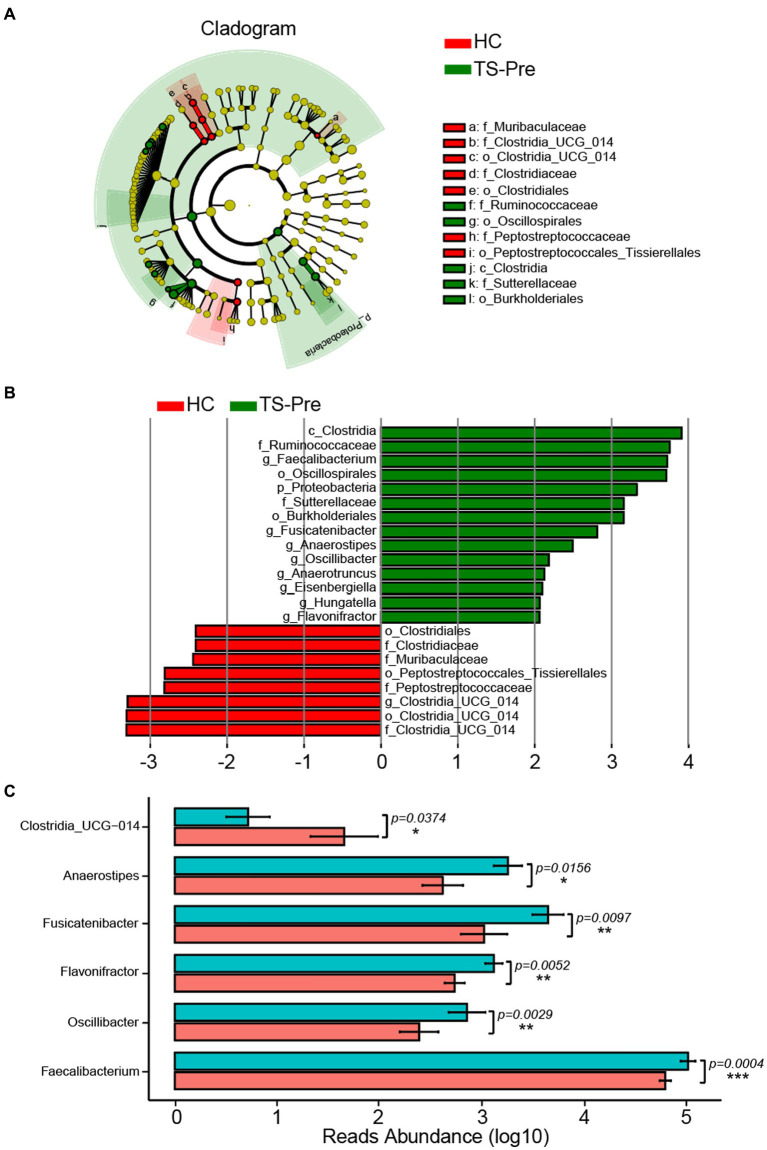
Marker Genera of gut microbiota between the HC group and the TS-Pre group. **(A)** The phylogenetic tree displayed the marker taxa according to subordinate relationship from phylum to species levels for these two groups. **(B)** Key altered phylotypes between both groups at different levels. **(C)** Significant differences in genera between the HC and the TS-Pre groups.

Due to the limitation of 16S rDNA amplicon pyrosequencing, we mainly focused on the downstream analysis at the genus level. The Wilcoxon rank sum test (LEfSe) (*p* < 0.05, LDA > 2) was used to identify the marker genera for different groups, and the TS-Pre group had a greater number of marker bacteria than the HC group (Figure 2). *Faecalibacterium, Hungatella, Oscillibacter, Flavonifractor, Fusicatenibacter, Anaerostipes, Anaerotruncus,* and *Eisenbergiella* were the marker genera for the TS-Pre group, whereas only Clostridia_UCG_014 was the marker genus for HC group (Figure 2B). In addition, we also acquired statistical significances of the differential genera, and the *p* values of *Faecalibacterium, Oscillibacter, Flavonifractor, Fusicatenibacter, Anaerostipes,* and *Clostridia_UCG_014* were 0.0004, 0.0029, 0.0052, 0.0097, 0.0156, and 0.0374, respectively (Figure 2C).

### Alterations of potential metabolic pathways in children with TS

**Figure 3:**
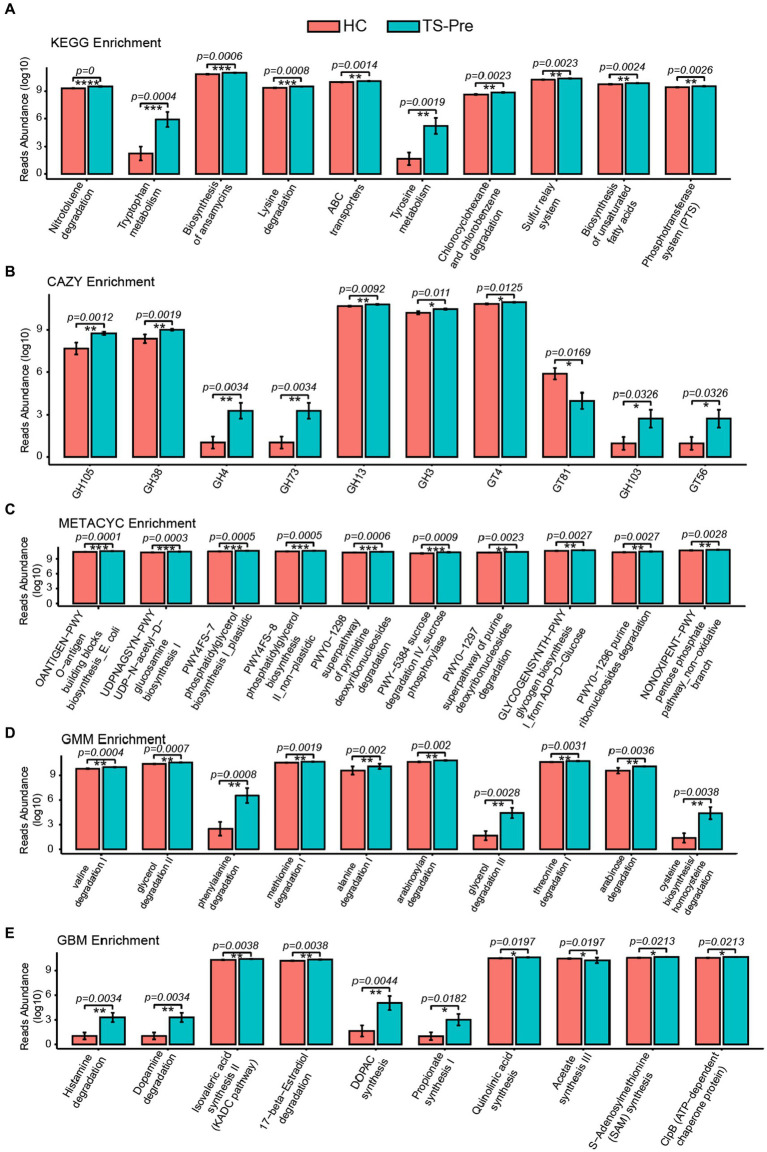
KEGG, CAZY, METACYC, GMM, and GBM were enriched by Picrust2 functional predictive analysis in the HC group and the TS-Pre group. The top 10 differential pathways in KEGG **(A)**, CAZY **(B)**, METACYC **(C)**, GMM **(D)**, and GBM **(E)** between both groups, respectively. Statistical analysis was performed by Univar Test. ^*^p < 0.05, ^**^p < 0.01, ^***^p < 0.001.

To explore potential functional alterations associated with the gut microbial changes in patients with TS, KEGG, CAZY, METACYC, GMM, and GBM module enrichment analysis were predicted by Picrust2 between the HC group and the TS-Pre group. The top 10 significant differences in KEGG, CAZY, METACYC, GMM, and GBM module enrichments between groups were shown in Figure 3. Noteworthy, as shown in Figure 3E, multiple GBM neurotransmitter modules related to neurodevelopmental disorders were abnormal in the TS-Pre group, such as histamine degradation, dopamine degradation, and DOPAC synthesis.

### Significant improvement was found in children with TS after the combinational physiotherapy

**Figure 4:**
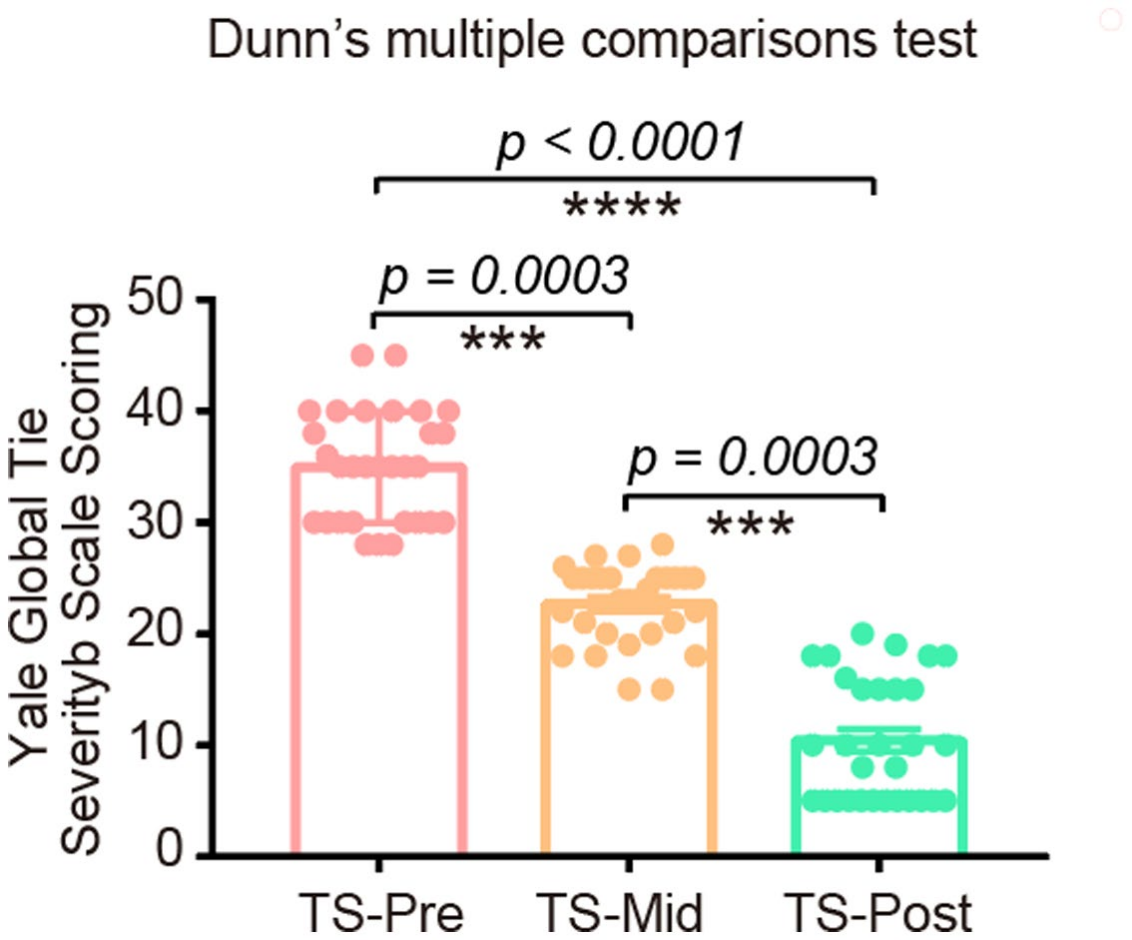
The YGTSS scores in TS patients before, after one course of, and after two courses of this combinational physiotherapy consisting of cranial electrotherapy stimulation plus electrodermal biofeedback training. These scores were significantly decreased after this treatment.

Among 32 children with TS, 2 were treatment-naïve, and 30 were treated with this combinational physiotherapy. As the YGTSS score is an important indicator to evaluate patients’ condition, we recorded the YGTSS scores for every patient before, after the first 10-day course, and after two 10-day courses of this treatment. Compared with patients before the combinational physiotherapy, patients after this treatment presented much lower YGTSS scores (Figure 4). These patients’ conditions improved dramatically, including the alleviation of motor tics and vocal tics, which was confirmed by both their guardians and primary physicians. During the treatment process and follow-up period, no distinct adverse reactions were observed in any of the children.

### Gut microbial composition in patient after the combinational physiotherapy as different from that in patients without any treatment

**Figure 5:**
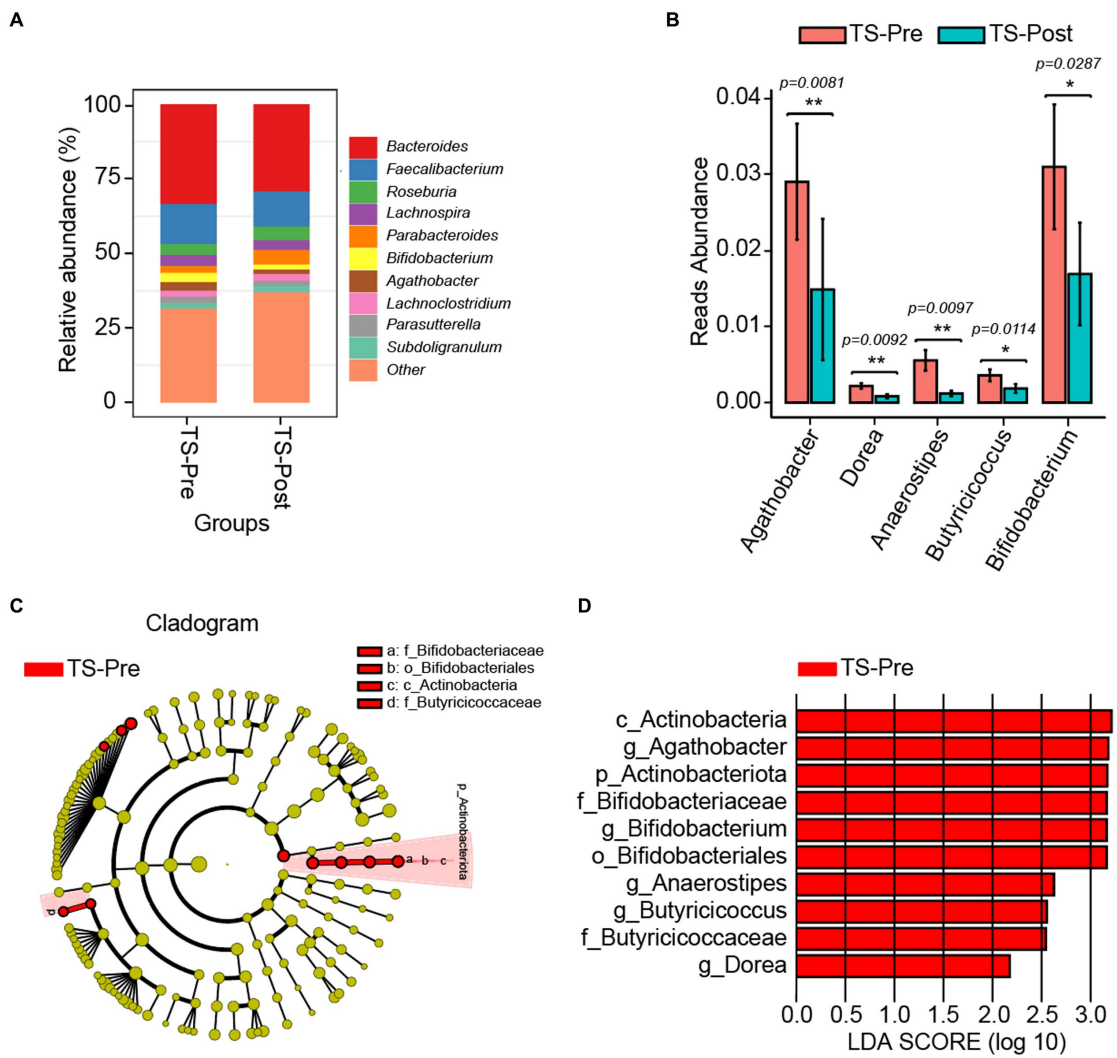
Gut microbial composition in TS patients after two courses of this combinational physiotherapy (TS-Post, *n* = 17) was different from that in patients before this treatment (TS-Pre, *n* = 32). **(A)** The relative abundance histograms of all genera in the TS-Pre and the TS-Post groups. Different colors represented the top 10 shared genera, and “other” represented the remaining genera with less relative abundance. **(B)** Significant differences in genera between the TS-Pre group and the TS-Post group. **(C)** The phylogenetic tree displayed the marker taxa according to subordinate relationship from phylum to species levels for both groups. **(D)** Key altered phylotypes between both groups in generic levels. ^*^p < 0.05, ^**^p < 0.01.

Due to the bidirectional communication between the gut and the brain, we wanted to explore the diversity and composition of gut microbiota in children with TS when their clinical symptoms were dramatically alleviated. 16S rDNA amplicon pyrosequencing was performed on fecal specimens, and no significant differences were observed in gut microbial diversity between patients before and after the combinational physiotherapy (Supplementary Figure S2). *Bacteroides, Faecalibacterium,* and *Roseburia* were the major components in the TS-Pre group, whereas *Bacteroides, Faecalibacterium,* and *Parabacteroides* in the TS-Post group (Figure 5A and Supplementary Figure S3). Five differential genera were identified between both groups by Kruskal–Wallis test and the physiotherapy-treated children had lower abundances of *Agathobacter, Dorea, Anaerostipes, Butyricicoccus,* and *Bifidobacterium* (Figure 5B).

In addition, we also explored the marker bacteria in both groups via LEfSe (*p* < 0.05, LDA > 2). At the genus level, *Agathobacter, Dorea, Anaerostipes, Butyricicoccus,* and *Bifidobacterium* were the marker genera in the TS-Pre group, whereas no marker genus was found in the TS-Post group, partly due to the relatively small sample size with 17 (Figures 5C,D).

### Potential metabolic pathways in patient after this combinational physiotherapy was different from that in patients before this treatment

**Figure 6:**
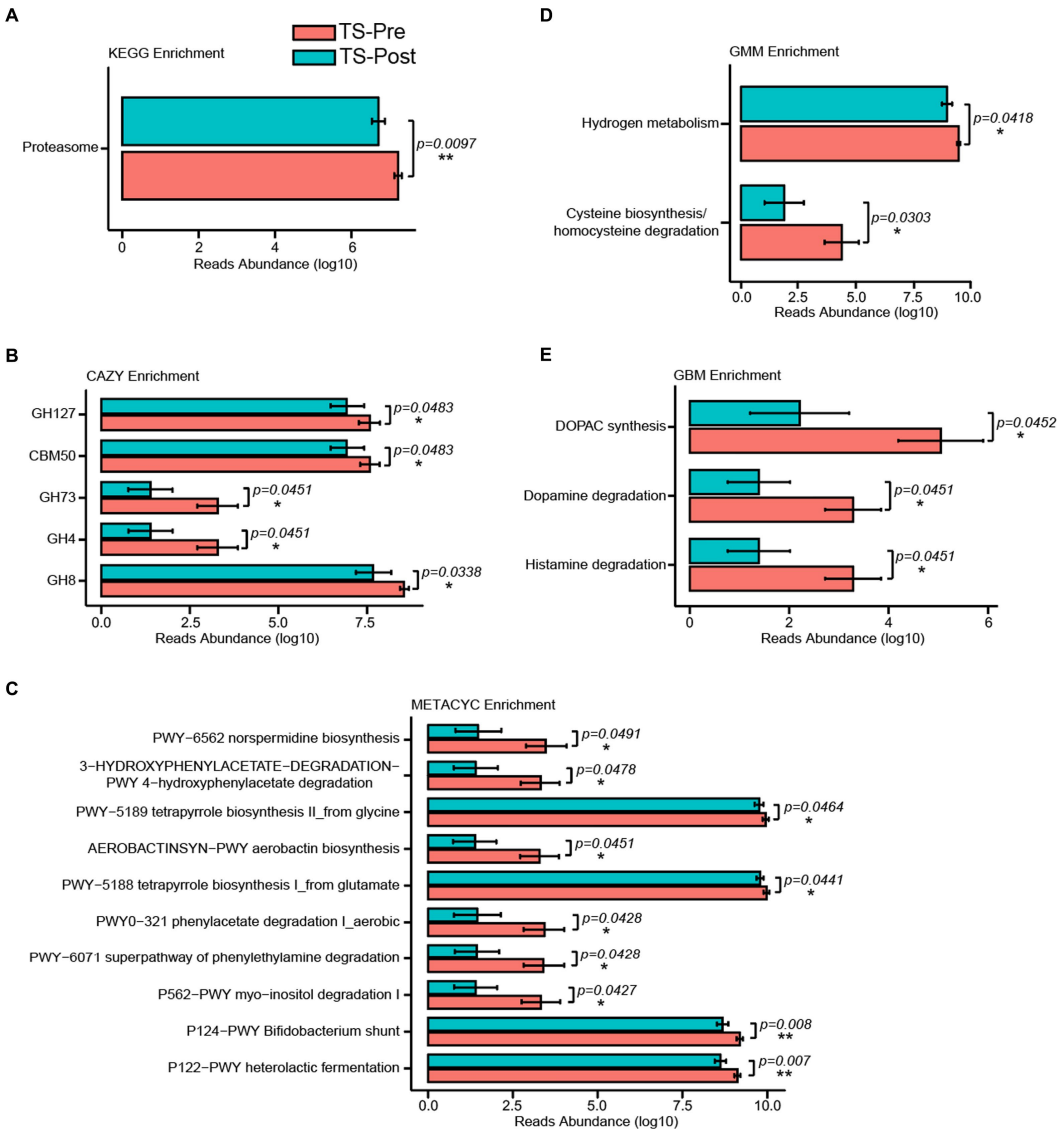
KEGG, CAZY, METACYC, GMM, and GBM were enriched by Picrust2 functional predictive analysis in the TS-Pre group and the TS-Post group. The KEGG **(A)**, CAZY **(B)**, METACYC **(C)**, GMM **(D)**, and GBM **(E)** pathways presented 1, 5, 10, 2, and 3 significant differences between both groups, respectively. Statistical analysis was performed by Univar Test. ^*^p < 0.05, ^**^p < 0.01.

To explore the potential metabolic pathways associated with the gut microbial alterations in patients after this combinational physiotherapy, KEGG, CAZY, METACYC, GMM, and GBM modules enrichment analysis were also predicted by Picrust2 between patients before and after this treatment. One KEGG pathway was found between the TS-Pre group and the TS-Post group, which was Proteasome (*p* = 0.0097) (Figure 6A). Meanwhile, five, ten, and two different modules were observed in CAZY, METACYC, and GMM modules enrichment analysis, respectively (Figures 6B–D). Noteworthy, as shown in Figure 6E, three different GBM neurotransmitter modules between both groups were identified, which were Histamine degradation, Dopamine degradation, and DOPAC synthesis.

## Discussion

This preliminary study not only aimed to depict the gut microbial profiles of children with TS, but also aimed to explore the potential clinical effects of this combinational physiotherapy and its influences on the gut microbiome in children with TS. Our results indicated that the gut microbial composition in patients was different from that in healthy controls, with much higher abundances of the genera *Faecalibacterium, Hungatella, Oscillibacter, Flavonifractor, Fusicatenibacter, Anaerostipes, Anaerotruncus,* and *Eisenbergiella*. Moreover, we also found that this treatment could significantly diminish tic activity in patients, whose potential positive effects were first reported in TS. Lastly, different gut microbial composition was also observed between TS patients before and after the combinational physiotherapy, with lower abundances of the genera *Agathobacter, Dorea, Anaerostipes, Butyricicoccus,* and *Bifidobacterium* in patients after this treatment, partly due to the bidirectional communication of the gut-brain axis (39, 40). However, our results should be treated with caution due to the rudimentariness of this study.

*Flavonifractor* degrades flavonoid quercetin (a flavonoid with antioxidant and anti-inflammatory properties), increasing oxidative stress and inflammation (41). Interestingly, our study first reported the enrichment of *Flavonifractor* in patients with TS, which was consistent with the existing assumption that pro-inflammatory pathogenesis existed in the occurrence and progression of TS (7). Last year, Eicher and Mohajeri reported that patients with seven brain-related diseases (attention deficit hyperactivity disorder, autism spectrum disorder, schizophrenia, Alzheimer’s disease, Parkinson’s disease, major depressive disorder, and bipolar disorder) presented higher abundances of pro-inflammatory bacteria (*Alistipes, Eggerthella, Flavonifractor*) in comparison with healthy controls (42). However, the abundance of *Flavonifractor* did not show a significant decrease between patients before and after the combinational physiotherapy in our study. Partly because it is less rigorous to conclude that *Flavonifractor* induced oxidative stress and inflammation in the host, and further researches about the direct associations between *Flavonifractor* and oxidative stress are essential. In addition, although several marker genera were identified in the TS-Pre group, no marker genera were found in the TS-Post group, partly due to the relatively small sample size. Moreover, the characteristic bacteria have not been established because of the short therapeutic period of 20 days. However, no researchers have reported the gut microbial alterations related to this treatment in patients with TS. Thus, these results should be treated cautiously and needed to be verified by large and multi-centric samples.

It is interesting to explore the functional changes associated with gut microbial alterations between healthy controls and patients with TS. In this study, multiple GBM neurotransmitter modules related to neurodevelopmental disorders were significantly increased in patients with TS, including Histamine degradation, Dopamine degradation, and DOPAC synthesis. Until now, several neurotransmitters produced by gut bacteria were associated with behavioral states and involved in the pathophysiology of TDs, such as dopamine and histamine (43, 44). Moreover, the abnormal dopamine pathway was studied well in TDs, and dopamine receptor antagonist (DRA) was used to suppress tics (45, 46). Published studies also have reported that DRA could alter the diversity and composition of gut microbiota in children with or without TDs (7, 47). Expectedly, the metabolic pathways of DOPAC synthesis, dopamine degradation, and histamine degradation were all decreased in patients after the combinational physiotherapy compared with patients before the treatment in this study, indicating that improved clinical symptoms associated with emotion might be accompanied by an improvement of the intestinal microenvironment. Signals from the brain could affect sensory, motor and secretory modalities of the gastrointestinal tract, and many studies have identified the influence of stress on gut microbiota, including physical and psychological stressors (48). A decreased abundance of *Turicibacter* spp. and an increased abundance of *Ruminococcus gnavus* were observed by exercise-induced stress in mouse cecum, and both of them play important roles in enteral mucus degradation and immune function (49). Exposure to a prolonged restraint stressor gave rise to an overgrowth of facultatively anaerobic microbiota, and a decreased diversity and abundance of cecal microbiota in mice, which could be partly explained by intestinal inflammation related to the bacterial abundance variation in the family Porphyromonadaceae (50). Exposure to a social stressor (i.e., social disruption) also could significantly change gut microbial compositions, and three changed bacterial genera (i.e., Coprococcus, Pseudobutyrivibrio, and Dorea) were dramatically positively correlated with the circulating levels of IL-6 and MCP-1 (51). Interestingly, in comparison with patients before the combinational physiotherapy, patients after this treatment also presented a significant reduction in the abundance of genera Dorea, indicating potential decreases in circulating cytokine.

Except for overcoming the side effects of medication and the long treatment period for psychological treatment, our combinational physiotherapy also costs less than other noninvasive techniques (e.g., transcranial magnetic stimulation) (10, 11). In this study, we did observe a significant reduction in tics according to children’s YGTSS scores and guardians’ positive reports. As far as we know, this is the first study to combine CES with electrodermal biofeedback training to treat TS and acquire a positive effect. In the aspect of neurophysiology, the intervention of CES could improve neural dysfunction and relieve tic symptoms for TS patients by regulating their brain activity. In comparison with TS adolescents before CES, adolescents after CES presented a suppression in the functional activity and connectivity of motor pathways and an increase in the control portions within the cortico-striato-thalamo-cortical (CSTC) circuit, indicating the recovery of TS might benefit from the normalization of intrinsic neural circuits (17). In addition, both the activity of the medial orbitofrontal cortex and the amplitude of slow cortical potentials can be regulated by electrodermal biofeedback (52, 53). Since the dysfunctions of the orbitofrontal cortex and the low preparatory motor potentials were observed in TS (54–57), it was reasonable to infer that the electrodermal biofeedback could affect neural circuits related to the motor tics by regulating cortical excitability. However, Nagai et al. found that electrodermal biofeedback could briefly decrease TS patients’ sympathetic activity, but not reduce their tics (53). Partly because of the high dropout rate occurring in their active-biofeedback group (53). On the other hand, in our study, a 60-min CES treatment was followed by a 30-min duration of electrodermal biofeedback training twice a day lasting for 20 days, whose training approaches and frequencies were different from the above study only including a 30-min duration of the biofeedback session once a week. However, the functional brain imaging data was not included in this study due to a lack of funds, and we have applied for another funding to perform a multicenter study with more participants and more comprehensive measures.

Several limitations should be noted in this study. Firstly, it is difficult to generalize our findings to popularity due to the relatively small sample size with homogeneous children. Ideally, data acquired from larger and multi-centric training samples are required to verify our findings. Second, the gut microbial composition was affected by fecal consistency (58), which was ignored by us in this study. However, according to our knowledge, all participants defecated smoothly and had no diarrhea during the study. Thirdly, CES treatment is regarded as a safe neuro-medical treatment for TS, which is mostly based on studies focused on anxiety, depression, or insomnia (59). Finally, functional brain imaging of patients before and after this combinational physiotherapy was not recorded, which might contribute to revealing functional activity and connectivity among different brain areas in TS (60–62). Therefore, we recommend that readers interpreted our findings with caution due to these above limitations.

## Conclusion

In short, children with TS showed a cognizable gut microbial profile, and certain enriched bacteria (e.g., Flavonifractor) with pro-inflammatory potentials might induce neuroinflammatory responses. CES plus electrodermal biofeedback training could significantly diminish tic symptoms, and different gut microbial compositions were also observed between TS patients before and after this combinational physiotherapy, indicating the existence of bidirectional communication of the gut-brain axis in TS. But studies on the gut microbial characteristics in TS patients, the influences of gut microbiota on tic severity, the efficacy and safety of this treatment, and the bidirectional regulatory mechanism between brain signals and gut microbiota in TS still need to be explored.

## Data availability statement

The original contributions presented in the study are publicly available. This data can be found here: NCBI BioProject database PRJNA909064.

## Ethics statement

The studies involving humans were approved by the Ethics Committee of the Xiangyang No. 1 People’s Hospital, Hubei University of Medicine (Xiangyang, China). The studies were conducted in accordance with the local legislation and institutional requirements. Written informed consent for participation in this study was provided by the participants' legal guardians/next of kin.

## Author contributions

HJ and CB designed, funded, and supervised the study, revised this manuscript, approved the final version of the manuscript on behalf of MeW, HP, MiW, ZL, and YX. CB, MeW, and HP recruited eligible subjects, collected their samples, and conducted YGTSS scores. MiW, ZL, YX, and HJ worked on the analysis and interpretation of data. HJ drafted the manuscript. All authors contributed to the article and approved the submitted version.

## Funding

This research was supported by the Science and Technology project of Xiangyang (No. 2020YL32).

## Conflict of interest

MiW, ZL, YX, and HJ were employed by Shanghai Biotecan Pharmaceuticals Co., Ltd.

The remaining authors declare that the research was conducted in the absence of any commercial or financial relationships that could be construed as a potential conflict of interest.

## Publisher’s note

All claims expressed in this article are solely those of the authors and do not necessarily represent those of their affiliated organizations, or those of the publisher, the editors and the reviewers. Any product that may be evaluated in this article, or claim that may be made by its manufacturer, is not guaranteed or endorsed by the publisher.

## Footnotes

^1^http://huttenhower.sph.harvard.edu/GraPhlAn

## References

[1] Cohen SC Leckman JF Bloch MH. Clinical assessment of Tourette syndrome and tic disorders. Neurosci Biobehav Rev. (2013) 37:997–1007. doi: 10.1016/j.neubiorev.2012.11.013 23206664 PMC3674220

[2] Knight T Steeves T Day L Lowerison M Jette N Pringsheim T. Prevalence of tic disorders: a systematic review and Meta-analysis. Pediatr Neurol. (2012) 47:77–90. doi: 10.1016/j.pediatrneurol.2012.05.002 22759682

[3] Yang C Zhang L Zhu P Zhu C Guo Q. The prevalence of tic disorders for children in China: a systematic review and Meta-analysis. Medicine (Baltimore). (2016) 95:e4354. doi: 10.1097/MD.0000000000004354 27472724 PMC5265861

[4] Hartmann A Worbe Y Black KJ. Tourette syndrome research highlights from 2017. F1000Res. (2018) 7:1122. doi: 10.12688/f1000research.15558.1 30210792 PMC6107994

[5] Liang S Wu X Jin F. Gut-brain psychology: rethinking psychology from the microbiota-gut-brain Axis. Front Integr Neurosci. (2018) 12:33. doi: 10.3389/fnint.2018.00033 30271330 PMC6142822

[6] Mayer EA Nance K Chen S. The gut-brain Axis. Annu Rev Med. (2022) 73:439–53. doi: 10.1146/annurev-med-042320-014032 34669431

[7] Xi W Gao X Zhao H Luo X Li J Tan X. Depicting the composition of gut microbiota in children with tic disorders: an exploratory study. J Child Psychol Psychiatry. (2021) 62:1246–54. doi: 10.1111/jcpp.13409 33738808

[8] Strandwitz P Kim KH Terekhova D Liu JK Sharma A Levering J. Gaba-modulating Bacteria of the human gut microbiota. Nat Microbiol. (2019) 4:396–403. doi: 10.1038/s41564-018-0307-3 30531975 PMC6384127

[9] Yunes RA Poluektova EU Dyachkova MS Klimina KM Kovtun AS Averina OV. Gaba production and structure of Gadb/Gadc genes in Lactobacillus and Bifidobacterium strains from human microbiota. Anaerobe. (2016) 42:197–204. doi: 10.1016/j.anaerobe.2016.10.011 27794467

[10] Ueda K Black KJ. A comprehensive review of tic disorders in children. J Clin Med. (2021) 10:2479. doi: 10.3390/jcm10112479 34204991 PMC8199885

[11] Andren P Jakubovski E Murphy TL Woitecki K Tarnok Z Zimmerman-Brenner S. European clinical guidelines for Tourette syndrome and other tic disorders-version 2.0. Part ii: psychological interventions. Eur Child Adolesc Psychiatry. (2022) 31:403–23. doi: 10.1007/s00787-021-01845-z 34313861 PMC8314030

[12] Hsu CW Wang LJ Lin PY. Efficacy of repetitive transcranial magnetic stimulation for Tourette syndrome: a systematic review and Meta-analysis. Brain Stimul. (2018) 11:1110–8. doi: 10.1016/j.brs.2018.06.002 29885862

[13] Zaghi S Acar M Hultgren B Boggio PS Fregni F. Noninvasive brain stimulation with low-intensity electrical currents: putative mechanisms of action for direct and alternating current stimulation. Neuroscientist. (2010) 16:285–307. doi: 10.1177/1073858409336227 20040569

[14] Bystritsky A Kerwin L Feusner J. A pilot study of cranial electrotherapy stimulation for generalized anxiety disorder. J Clin Psychiatry. (2008) 69:412–7. doi: 10.4088/jcp.v69n0311 18348596

[15] Kirsch DL Nichols F. Cranial electrotherapy stimulation for treatment of anxiety, depression, and insomnia. Psychiatr Clin North Am. (2013) 36:169–76. doi: 10.1016/j.psc.2013.01.006 23538086

[16] McClure D Greenman SC Koppolu SS Varvara M Yaseen ZS Galynker II. A pilot study of safety and efficacy of cranial electrotherapy stimulation in treatment of bipolar ii depression. J Nerv Ment Dis. (2015) 203:827–35. doi: 10.1097/NMD.0000000000000378 26414234 PMC4892785

[17] Qiao J Weng S Wang P Long J Wang Z. Normalization of intrinsic neural circuits governing Tourette's syndrome using cranial electrotherapy stimulation. IEEE Trans Biomed Eng. (2015) 62:1272–80. doi: 10.1109/TBME.2014.2385151 25546850

[18] Ya HX Li GH Zhang JJ. A six-month clinical observation of cranial electrotherapy stimulation for children's refractory Tourette syndrome. J Clin Psychiatry. (2015) 5:40–1. (Chinese Journal).

[19] Wu WJ Wang Y Cai M Chen YH Zhou CH Wang HN. A double-blind, randomized, sham-controlled study of cranial electrotherapy stimulation as an add-on treatment for tic disorders in children and adolescents. Asian J Psychiatr. (2020) 51:101992. doi: 10.1016/j.ajp.2020.101992 32145674

[20] Wu CJ Chen YH. A control study of cranial electrotherapy stimulation and aripiprazole treatment for tic disorders in children. Chin J Child Health Care. (2016) 24:576–8. (Chinese Journal).

[21] Nagai Y Cavanna A Critchley HD. Influence of sympathetic autonomic arousal on tics: implications for a therapeutic behavioral intervention for Tourette syndrome. J Psychosom Res. (2009) 67:599–605. doi: 10.1016/j.jpsychores.2009.06.004 19913664

[22] Nagai Y Cavanna AE Critchley HD Stern JJ Robertson MM Joyce EM. Biofeedback treatment for Tourette syndrome: a preliminary randomized controlled trial. Cogn Behav Neurol. (2014) 27:17–24. doi: 10.1097/WNN.0000000000000019 24674962

[23] Cussotto S Sandhu KV Dinan TG Cryan JF. The neuroendocrinology of the microbiota-gut-brain Axis: a Behavioural perspective. Front Neuroendocrinol. (2018) 51:80–101. doi: 10.1016/j.yfrne.2018.04.002 29753796

[24] Berthoud HR. Vagal and hormonal gut-brain communication: from satiation to satisfaction. Neurogastroenterol Motil. (2008) 20 Suppl 1:64–72. doi: 10.1111/j.1365-2982.2008.01104.x 18402643 PMC3617963

[25] Konturek SJ Konturek JW Pawlik T Brzozowski T. Brain-gut Axis and its role in the control of food intake. J Physiol Pharmacol. (2004) 55:137–54. 15082874

[26] Tache Y Vale W Rivier J Brown M. Brain regulation of gastric secretion: influence of neuropeptides. Proc Natl Acad Sci U S A. (1980) 77:5515–9. doi: 10.1073/pnas.77.9.5515 6159649 PMC350092

[27] Agusti A Garcia-Pardo MP Lopez-Almela I Campillo I Maes M Romani-Perez M. Interplay between the gut-brain Axis, obesity and cognitive function. Front Neurosci. (2018) 12:155. doi: 10.3389/fnins.2018.00155 29615850 PMC5864897

[28] Carabotti M Scirocco A Maselli MA Severi C. The gut-brain Axis: interactions between enteric microbiota, central and enteric nervous systems. Ann Gastroenterol. (2015) 28:203–9. 25830558 PMC4367209

[29] Sarkar A Lehto SM Harty S Dinan TG Cryan JF Burnet PWJ. Psychobiotics and the manipulation of Bacteria-gut-brain signals. Trends Neurosci. (2016) 39:763–81. doi: 10.1016/j.tins.2016.09.002 27793434 PMC5102282

[30] Zhao H Shi Y Luo X Peng L Yang Y Zou L. The effect of fecal microbiota transplantation on a child with Tourette syndrome. Case Rep Med. (2017) 2017:6165239. doi: 10.1155/2017/6165239 29666652 PMC5865276

[31] Zhao HJ Luo X Shi YC Li JF Pan F Ren RR. The efficacy of fecal microbiota transplantation for children with Tourette syndrome: a preliminary study. Front Psych. (2020) 11:554441. doi: 10.3389/fpsyt.2020.554441 PMC7793740 33424650

[32] Li H Wang Y Zhao C Liu J Zhang L Li A. Fecal transplantation can alleviate tic severity in a Tourette syndrome mouse model by modulating intestinal Flora and Promoting serotonin secretion. Chin Med J. (2022) 135:707–13. doi: 10.1097/CM9.0000000000001885 35288507 PMC9276343

[33] Wang Z. Effect of pediatric massage combined with acupuncture on gut microbiota structure of children with Tourette syndrome. Hohhot, Inner Mongolia autonomous Region, China: Inner Mongolia Medical University (2021) MA thesis.

[34] Caporaso JG Kuczynski J Stombaugh J Bittinger K Bushman FD Costello EK. Qiime allows analysis of high-throughput community sequencing data. Nat Methods. (2010) 7:335–6. doi: 10.1038/nmeth.f.303 20383131 PMC3156573

[35] Gill SR Pop M Deboy RT Eckburg PB Turnbaugh PJ Samuel BS. Metagenomic analysis of the human distal gut microbiome. Science. (2006) 312:1355–9. doi: 10.1126/science.1124234 16741115 PMC3027896

[36] Rognes T Flouri T Nichols B Quince C Mahe F. Vsearch: a versatile open source tool for metagenomics. PeerJ. (2016) 4:e2584. doi: 10.7717/peerj.2584 27781170 PMC5075697

[37] Segata N Izard J Waldron L Gevers D Miropolsky L Garrett WS. Metagenomic biomarker discovery and explanation. Genome Biol. (2011) 12:R60. doi: 10.1186/gb-2011-12-6-r60 21702898 PMC3218848

[38] Langille MG Zaneveld J Caporaso JG McDonald D Knights D Reyes JA. Predictive functional profiling of microbial communities using 16s Rrna marker gene sequences. Nat Biotechnol. (2013) 31:814–21. doi: 10.1038/nbt.2676 23975157 PMC3819121

[39] Foster JA McVey Neufeld KA. Gut-brain Axis: how the microbiome influences anxiety and depression. Trends Neurosci. (2013) 36:305–12. doi: 10.1016/j.tins.2013.01.005 23384445

[40] Vuong HE Hsiao EY. Emerging roles for the gut microbiome in autism Spectrum disorder. Biol Psychiatry. (2017) 81:411–23. doi: 10.1016/j.biopsych.2016.08.024 27773355 PMC5285286

[41] Carlier JP Bedora-Faure M K'Ouas G Alauzet C Mory F. Proposal to unify *Clostridium Orbiscindens* Winter et al. 1991 and *Eubacterium Plautii* (Seguin 1928) Hofstad and Aasjord 1982, with description of *Flavonifractor Plautii* gen. Nov., comb. Nov., and reassignment of *Bacteroides Capillosus* to *Pseudoflavonifractor Capillosus* gen. Nov., comb. Nov. Int J Syst Evol Microbiol. (2010) 60:585–90. doi: 10.1099/ijs.0.016725-0 19654357

[42] Eicher TP Mohajeri MH. Overlapping mechanisms of action of brain-active Bacteria and bacterial metabolites in the pathogenesis of common brain diseases. Nutrients. (2022) 14:2661. doi: 10.3390/nu14132661 35807841 PMC9267981

[43] Gasbarri A Pompili A Packard MG Tomaz C. Habit learning and memory in mammals: behavioral and neural characteristics. Neurobiol Learn Mem. (2014) 114:198–208. doi: 10.1016/j.nlm.2014.06.010 24981854

[44] Yael D Vinner E Bar-Gad I. Pathophysiology of tic disorders. Mov Disord. (2015) 30:1171–8. doi: 10.1002/mds.26304 26179434

[45] Felling RJ Singer HS. Neurobiology of Tourette syndrome: current status and need for further investigation. J Neurosci. (2011) 31:12387–95. doi: 10.1523/JNEUROSCI.0150-11.2011 21880899 PMC6703258

[46] Jankovic J. Treatment of tics associated with Tourette syndrome. J Neural Transm (Vienna). (2020) 127:843–50. doi: 10.1007/s00702-019-02105-w 31955299

[47] Bahr SM Tyler BC Wooldridge N Butcher BD Burns TL Teesch LM. Use of the second-generation antipsychotic, risperidone, and secondary weight gain are associated with an altered gut microbiota in children. Transl Psychiatry. (2015) 5:e652. doi: 10.1038/tp.2015.135 26440540 PMC4930121

[48] Molina-Torres G Rodriguez-Arrastia M Roman P Sanchez-Labraca N Cardona D. Stress and the gut microbiota-brain Axis. Behav Pharmacol. (2019) 30:187–200. doi: 10.1097/FBP.0000000000000478 30844962

[49] Clark A Mach N. Exercise-induced stress behavior, gut-microbiota-brain Axis and diet: a systematic review for athletes. J Int Soc Sports Nutr. (2016) 13:43. doi: 10.1186/s12970-016-0155-6 27924137 PMC5121944

[50] Lupp C Robertson ML Wickham ME Sekirov I Champion OL Gaynor EC. Host-mediated inflammation disrupts the intestinal microbiota and promotes the overgrowth of Enterobacteriaceae. Cell Host Microbe. (2007) 2:204. doi: 10.1016/j.chom.2007.08.002 18030708

[51] Bailey MT Dowd SE Galley JD Hufnagle AR Allen RG Lyte M. Exposure to a social stressor alters the structure of the intestinal microbiota: implications for stressor-induced immunomodulation. Brain Behav Immun. (2011) 25:397–407. doi: 10.1016/j.bbi.2010.10.023 21040780 PMC3039072

[52] Nagai Y Critchley HD Featherstone E Trimble MR Dolan RJ. Activity in ventromedial prefrontal cortex Covaries with sympathetic skin conductance level: a physiological account of a "default mode" of brain function. NeuroImage. (2004) 22:243–51. doi: 10.1016/j.neuroimage.2004.01.019 15110014

[53] Nagai Y Critchley HD Rothwell JC Duncan JS Trimble MR. Changes in cortical potential associated with modulation of peripheral sympathetic activity in patients with epilepsy. Psychosom Med. (2009) 71:84–92. doi: 10.1097/PSY.0b013e31818f667c 19075039

[54] George MS Trimble MR Costa DC Robertson MM Ring HA Ell PJ. Elevated frontal cerebral blood flow in Gilles De La Tourette syndrome: a 99tcm-Hmpao Spect study. Psychiatry Res. (1992) 45:143–51. doi: 10.1016/0925-4927(92)90022-v 1484907

[55] Worbe Y Gerardin E Hartmann A Valabregue R Chupin M Tremblay L. Distinct structural changes underpin clinical phenotypes in patients with Gilles De La Tourette syndrome. Brain. (2010) 133:3649–60. doi: 10.1093/brain/awq293 20959309

[56] O'Connor K Lavoie ME Robert M. Preparation and motor potentials in chronic tic and Tourette syndromes. Brain Cogn. (2001) 46:224–6. doi: 10.1016/s0278-2626(01)80071-3 11527335

[57] van Woerkom TC Fortgens C van de Wetering BJ Martens CM. Contingent negative variation in adults with Gilles De La Tourette syndrome. J Neurol Neurosurg Psychiatry. (1988) 51:630–4. doi: 10.1136/jnnp.51.5.630 3165440 PMC1033066

[58] Vandeputte D Falony G Vieira-Silva S Tito RY Joossens M Raes J. Stool consistency is strongly associated with gut microbiota richness and composition, enterotypes and bacterial growth rates. Gut. (2016) 65:57–62. doi: 10.1136/gutjnl-2015-309618 26069274 PMC4717365

[59] Gilula MF Barach PR. Cranial electrotherapy stimulation: a safe Neuromedical treatment for anxiety, depression, or insomnia. South Med J. (2004) 97:1269–70. doi: 10.1097/01.SMJ.0000136304.33212.06 15646771

[60] Mazzone L Yu S Blair C Gunter BC Wang Z Marsh R. An Fmri study of Frontostriatal circuits during the inhibition of eye blinking in persons with Tourette syndrome. Am J Psychiatry. (2010) 167:341–9. doi: 10.1176/appi.ajp.2009.08121831 20080981 PMC4295823

[61] Peterson BS Skudlarski P Anderson AW Zhang H Gatenby JC Lacadie CM. A functional magnetic resonance imaging study of tic suppression in Tourette syndrome. Arch Gen Psychiatry. (1998) 55:326–33. doi: 10.1001/archpsyc.55.4.326 9554428

[62] Bohlhalter S Goldfine A Matteson S Garraux G Hanakawa T Kansaku K. Neural correlates of tic generation in Tourette syndrome: an event-related functional Mri study. Brain. (2006) 129:2029–37. doi: 10.1093/brain/awl050 16520330

