# Supplementary material for: A preliminary study for the clinical effect of one combinational physiotherapy and its potential influence on gut microbial composition in children with Tourette syndrome
Source: Front Nutr. 2023 Sep 15;10:1184311. doi: 10.3389/fnut.2023.1184311 (PMC10541309; doi:10.3389/fnut.2023.1184311)
Supplement: Supplementary file 1 [file Data_Sheet_1.pdf]

A Preliminary Study for the Clinical Effect of One Combinational physiotherapy and its Potential Influence on Gut Microbial Composition in Children with Tourette Syndrome

Chun Bao[1,*], Meng Wei[1], Hongguo Pan[1], Ming Wen[2], Ziming Liu[2], Yue Xu[2], Huihui Jiang[2,*]

[1]Department of Child Healthcare, Xiang Yang No.1 People's Hospital Affiliated Hospital of Hubei University of Medicine, Xiangyang, China, [2]Zhangjiang Center for Translational Medicine, Shanghai Biotecan Pharmaceuticals Co., Ltd., Shanghai, China

[*]***Correspondence:***

Huihui Jiang

16111520022@fudan.edu.cn

Chun Bao

1732014736@qq.com

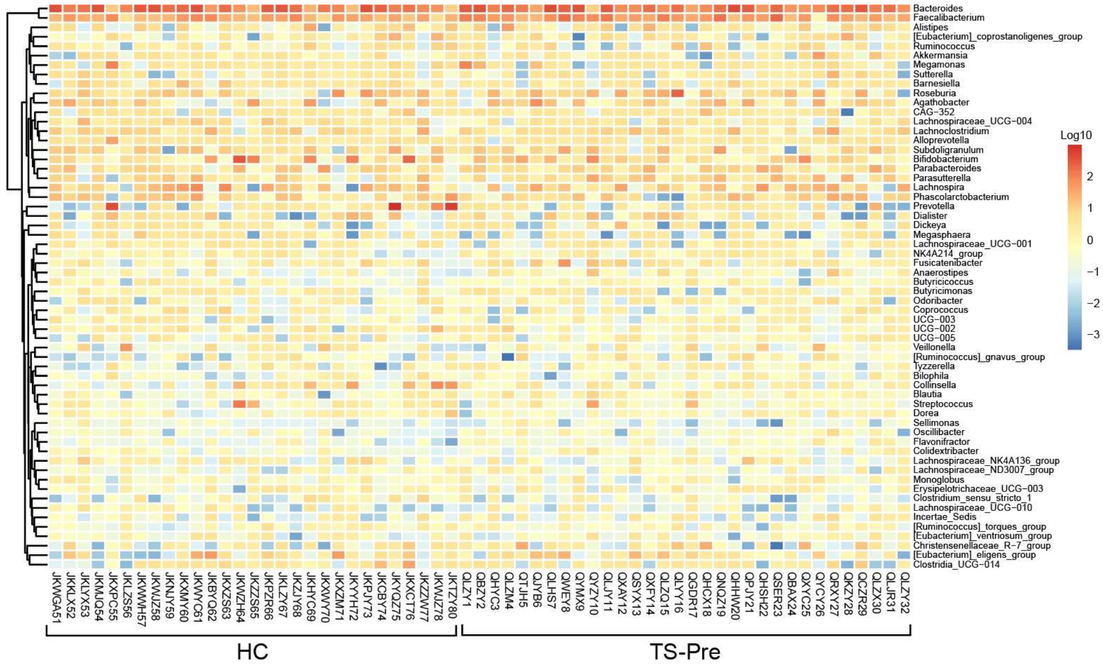

Supplementary figure 1. The top 60 genera were displayed according to the relative abundance of bacteria in healthy children and children with TS before receiving physiotherapy (TS-Pre).

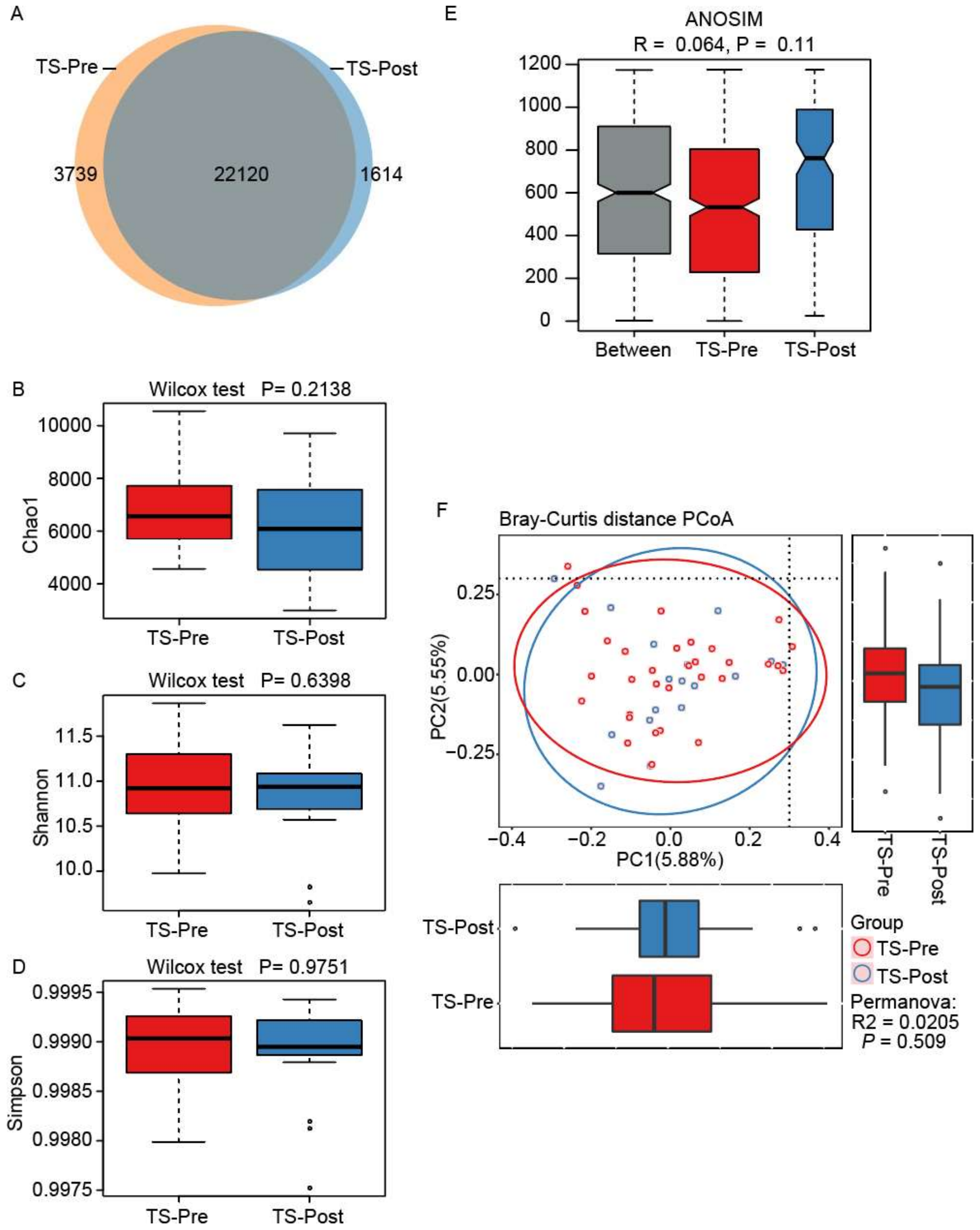

Supplementary figure 2. Diversity of Gut microbiota in children with TS before and after treated with physiotherapy. (A) 22120 OTUs were shared between groups. TS-Pre group had the unique OTUs with 3739, while the TS-Post group had the less with 1614. Chao1 (B), Shannon (C), and Simpson (D) were used to analyze the alpha diversity of gut microbiomes between the TS-Pre group (n = 32) and the TS-Post group (n = 17). (E) Analysis of similarity (ANOSIM) proved the difference between the groups was bigger than within the group ($p < 0.064$). (F) Beta diversity of gut microbiomes between the TS-Pre group and the TS-Post group was analyzed by principal coordinate analysis (PCoA) of the weighted UniFrac distance.

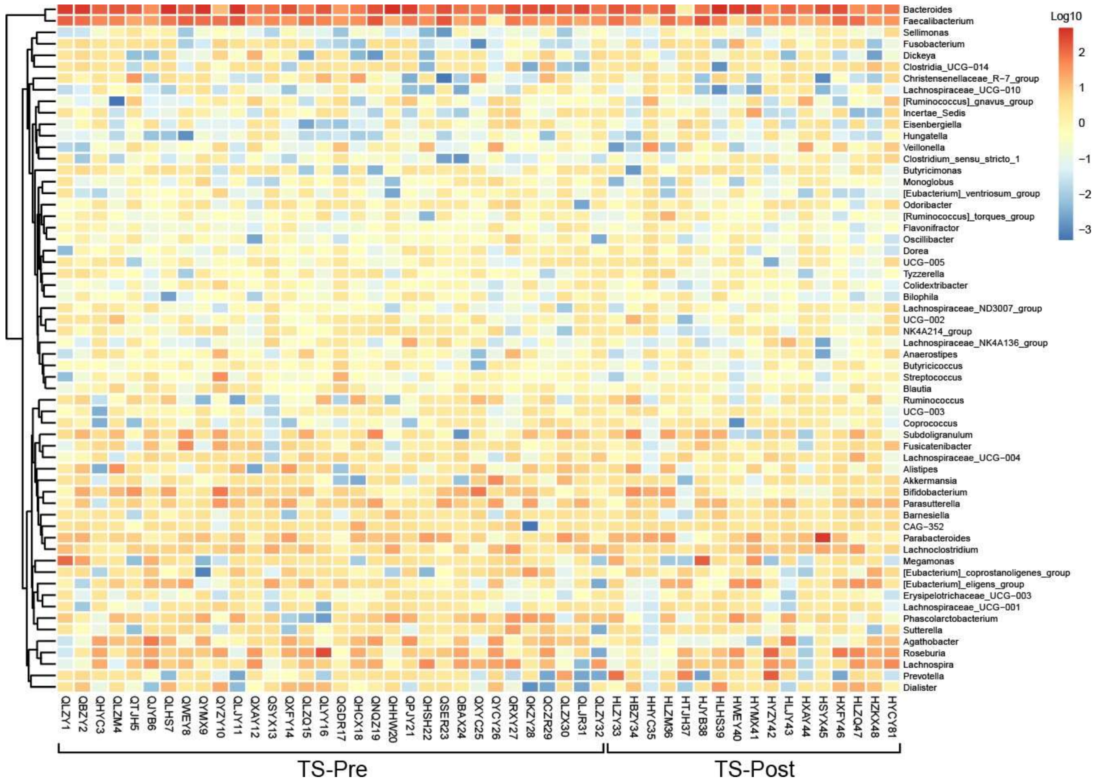

Supplementary figure 3. The top 60 genera were displayed according to the relative abundance of bacteria in children with TS before receiving the physiotherapy (TS-Pre) and after (TS-Post).
